# The mechanosensitive protein ANTXR1 is involved in maintaining cartilage homeostasis in post-traumatic osteoarthritis

**DOI:** 10.3389/fcell.2025.1625333

**Published:** 2025-09-03

**Authors:** Zhuangzhuang Miao, Junjie Liu, Pengfei Cheng, Yinghao Shen, Jiahang Tang, Jiajie Zhu, Weiwei Liu, Xuchang Zhou, Hongying Jing

**Affiliations:** ^1^ Graduate School, Harbin Sport University, Harbin, China; ^2^ College of science and technology, China Three Gorges University, Yichang, China; ^3^ School of Basic Medicine, China Three Gorges University, Yichang, China; ^4^ Graduate School, Kunming Medical University, Kunming, China; ^5^ School of Graduate Education, Shandong Sport University, Jinan, China; ^6^ School of Sport Medicine and Rehabilitation, Beijing Sport University, Beijing, China; ^7^ Department of Rehabilitation Medicine, the First Affiliated Hospital of Xiamen University, School of Medicine, Xiamen University, Xiamen, China

**Keywords:** osteoarthritis, ANTXR1, LRP6, treadmill exercise, anterior cruciate ligament osteoarthritis, anterior cruciate ligament injury

## Abstract

Post-traumatic osteoarthritis (PTOA) is a degenerative joint disease secondary to joint trauma, such as anterior cruciate ligament (ACL) injury. Our previous study found that the mechanosensitive protein ANTXR1 is involved in the regulation of bone homeostasis, however, whether ANTXR1 is involved in the regulation of cartilage homeostasis in PTOA is unclear. In this study, we focused on the expression of the mechanosensitive protein ANTXR1 in cartilage homeostasis and explored its role in the initiation and progression of PTOA. To investigate the effects of treadmill exercise on bone-cartilage homeostasis, we established models using different exercise intensities (20%, 40%, 60%, 80% of maximal oxygen uptake, VO_2_max). Histological staining (Hematoxylin and Eosin, HE) and micro-computed tomography (micro-CT) revealed that moderate-intensity exercise (40%–60% VO_2_max) effectively promoted bone-cartilage health compared to controls. Conversely, prolonged high-intensity exercise (8 weeks, 80% VO_2_max) demonstrated potentially deleterious effects on cartilage homeostasis. Immunofluorescence staining indicated that, compared to the sedentary group, both ANTXR1 protein expression in the knee cartilage was reduced across intervention groups, with expression levels progressively declining as exercise intensity increased. Notably, ANTXR1 and exhibited significantly higher expression in bone tissue compared to cartilage tissue. Bioinformatic analysis identified LRP6 as a potential target protein of ANTXR1. Furthermore, utilizing a post-traumatic osteoarthritis (PTOA) animal model, we explored the potential role of ANTXR1 in cartilage homeostasis and revealed that both ANTXR1 and LRP6 protein expression were significantly upregulated in the degenerating cartilage of PTOA rats. This study is the first to elucidate the dual roles of ANTXR1 in mechano-signaling and cartilage metabolic homeostasis, providing a molecular basis for developing ANTXR1-targeting inhibitors for PTOA treatment.

## 1 Introduction

Osteoarthritis (OA) is a degenerative disease of the joints characterized by progressive damage to articular cartilage and peripheral tissue lesions, including cartilage matrix degradation, osteophyte proliferation, synovial inflammation, subchondral osteosclerosis, and narrowing of the joint cavity, etc., (Hadzic and Beier, 2023). Risk factors for OA involve genetic predisposition, metabolic abnormalities, mechanical stress injuries, and gender differences (Al-Mahrouqi et al., 2018; Chaganti and Lane, 2011; Carlesso et al., 2017). Post-traumatic osteoarthritis (PTOA) is a subtype of OA, which refers to the destruction of joint structure due to acute or chronic injuries to the joints and surrounding tissues, such as fractures, cartilage injuries, ligament tears, and meniscus injuries, leading to degeneration of articular cartilage, osteophytes, and dysfunction (Kraus and Hsueh, 2024). Studies have reported that approximately 50% of patients with severe knee injuries develop PTOA within 10 years. However, the underlying pathogenesis has not been fully elucidated (Kornah et al., 2019). Cartilage homeostasis refers to the complex dynamic process of maintaining a balance between extracellular matrix (ECM) synthesis and degradation in articular cartilage under physiological conditions (Hao et al., 2025). During pathological changes in PTOA, mechanical stress, inflammatory responses, and abnormal biomechanics can activate chondrocytes toward a hypertrophic phenotype, leading to excessive ECM degradation, collagen fiber breakage, and chondrocyte apoptosis, ultimately disrupting cartilage homeostasis (Dilley et al., 2023).

Exercise, as a non-pharmacological intervention, has been widely demonstrated to improve joint function and delay cartilage degeneration in patients with OA (Fransen et al., 2015). Studies have confirmed that abnormal mechanical distribution in arthritic tissues (PTOA) can induce early subchondral sclerosis, osteochondrocyte necrosis, and apoptosis (Burr and Radin, 2003; Prasadam et al., 2013). In contrast, moderate mechanical loading promotes subchondral bone remodeling and maintains cartilage homeostasis through regulating chondrocyte metabolism and enhancing extracellular matrix (ECM) synthesis (Wu et al., 2025; Zhou et al., 2021). Low-density lipoprotein receptor-related protein 6 (LRP6), a member of the LDL receptor family, functions as a mechanosensitive protein and serves as a co-receptor for the canonical Wnt/β-catenin pathway. By binding to Frizzled receptors, LRP6 activates downstream signaling to drive osteoblast differentiation and bone formation. Study have shown that mechanical force-induced osteogenic responses in periodontal ligament stem cells (PDLSCs) are critically dependent on LRP6: Its knockdown via shRNA significantly reduced proliferation markers (e.g., PCNA) and osteogenic markers (ALP, RUNX2, OSX), suppressed ALP activity by 60%, and attenuated mineralization capacity. While current research predominantly focuses on mechanical regulation of osteogenesis, studies have shown that moderate mechanical loading can also maintain cartilage homeostasis by regulating chondrocyte metabolism and promoting ECM synthesis (Wu et al., 2025; Zhou et al., 2021). However, the specific mechanisms by which mechanical stress modulates cartilage homeostasis are not fully understood. Recently, the role of mechanosensitive proteins in cartilage metabolism has received increasing attention. Anthrax toxin consists of protective antigen (PA), edema factor (EF) and lethal factor (LF). Anthrax toxin receptor 1 (ANTXR1), also known as tumor endothelial marker (TEM8), is a type I transmembrane protein. The ANTXR1 protein contains a von Willebrand factor A (VWFA) structural domain that mediates toxin endocytosis by binding to the PA of anthrax toxin and exerts toxic effects in cells (Young and Collier, 2007). ANTXR1 interacts with the ECM and actin cytoskeleton to regulate cell adhesion, migration and spreading. Previous studies reported that ANTXR1 showed high expression in pathological microenvironments such as tumor vascular endothelium, cancer-associated fibroblasts (CAFs) and pericytes (Si et al., 2021). In addition, another study reported that ANTXR1 could be involved in regulating the Wnt/β-catenin pathway, which plays a key role in cartilage development and degeneration (Sung et al., 2025; Liu et al., 2021).

Our previous studies revealed that ANTXR1 can mediate exercise to promote bone formation (Liu et al., 2024). However, the role of mechanosensitive protein ANTXR1 in cartilage homeostasis in PTOA is unknown. The aim of this study was to investigate whether ANTXR1 expression in cartilage tissues responds to mechanical stress/exercise and whether ANTXR1 could be involved in regulating cartilage homeostasis in PTOA. This study contributes to a better understanding of the underlying mechanisms of exercise to alleviate OA and provides new targets for precision treatment of PTOA.

## 2 Materials and methods

**FIGURE 1:**
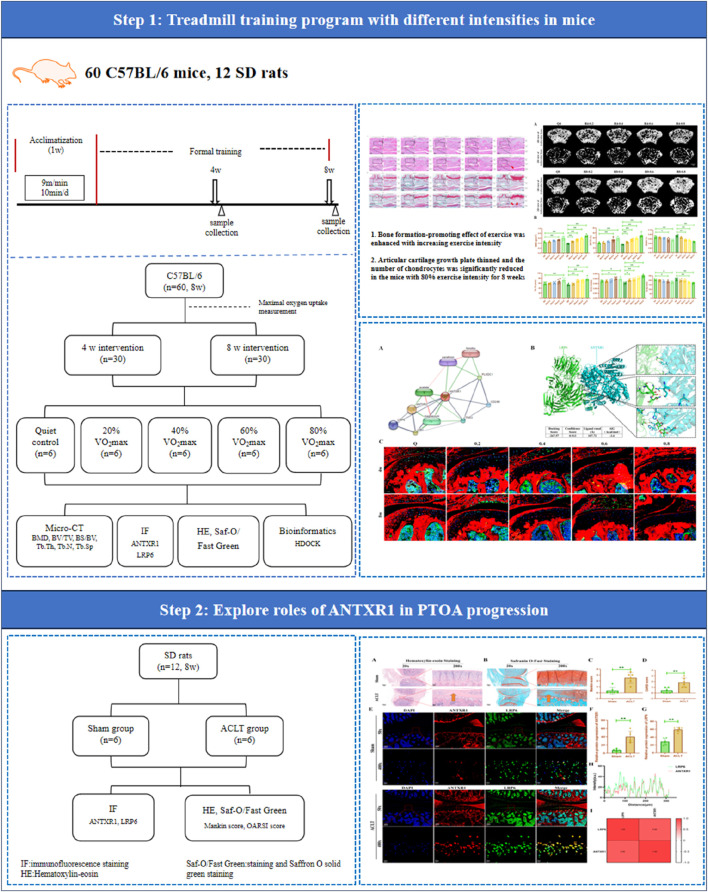
Flowchart of whole experiment.

All animal experiments in this study were reviewed and approved by the Ethics Committee of Exercise Science Experiments of Beijing Sport University (Approval No. 2023026A). The animals were housed in an SPF-class environment with temperature and relative humidity at (22 ± 2) °C and 55%–75%, respectively. All experimental animals were allowed to move freely in the cages. All animals were acclimatized for 1 week before starting formal experiments. The entire experimental procedure is illustrated in Figure 1.

### 2.1 Measurement of maximum oxygen uptake (VO_2_max)

After 1 week of acclimatization running in the intervention group, VO_2_max test was performed. To minimize any undue influence on the results, three C57BL/6 mice from each group (control and intervention; n = 6, see Section 2.2) were randomly selected for the VO_2_max test. Prior to testing, the selected mice underwent a 10-min warm-up exercise at a speed of 6 m/min with a 0° slope. Subsequently, the velocity was increased by 1 m/min every 3 minutes until maximum oxygen uptake was reached. The criteria for determining the numerical point of VO_2_max were: the mice could not continue to move on the running platform in the presence of electrical stimulation; the oxygen uptake reached a plateau and did not change with the increase in speed, or the growth rate of maximal oxygen uptake was less than 5% between the two levels. The running exercise program for each exercise group was determined based on the average maximal oxygen uptake (Schefer and Talan, 1996).

### 2.2 Treadmill training program with different intensities in mice

Sixty C57BL/6J male mice (8 weeks old) were purchased from Beijing Huafukang Biotechnology Co. Sixty mice were divided into a sedentary control group (n = 12) and a treadmill exercise group (n = 48). Half of the mice were sacrificed after 4 w of intervention, while the other half were sacrificed after 8 w of intervention. Mice in the treadmill exercise group underwent a 1-week acclimatization treadmill training prior to the formal experiment, followed by testing of maximal oxygen uptake, which was used to define the exercise intensity. Exercise regimens for each group of mice were determined based on 20%, 40%, 60%, and 80% of VO_2_max measurements. Therefore, 60 mice were randomly divided into 10 groups: 4w sedentary control group (Q4, n = 6), 4w treadmill exercise with 20% VO2max group (R4-0.2 group, n = 6), 4w treadmill exercise with 40% VO2max group (R4-0.4 group, n = 6), 4w treadmill exercise with 60% VO_2_max group (R4-0.6 group, n = 6), 4w treadmill exercise with 80% VO2max group (R4-0.8 group, n = 6), 8w sedentary control group (Q8, n = 6), 8w treadmill exercise with 20% VO2max group (R8-0.2 group, n = 6), 8w treadmill exercise with 40% VO2max group (R8-0.4 group, n = 6), 8w treadmill exercise with 60% VO2max group (R8-0.6 group, n = 6), 8w treadmill exercise with 80% VO_2_max group (R8-0.8 group, n = 6). Mice in the exercise group were trained on the treadmill 3 times per week for 1 h each according to their respective running regimens formulated through the corresponding maximal oxygen uptake (as shown in Table 1; Figure 1).

**TABLE 1:** Running exercise program for each group of mice.

Group	Acclimatization (1w)	Tradmill exercise	Time
Q	9 m/min,10 min/d	0 m/min,1 h/d,3d/w	4w/8w
20% VO_2_max		6.3 m/min,1 h/d,3d/w	4w/8w
40% VO_2_max		12.6 m/min,1 h/d,3d/w	4w/8w
60% VO_2_max		18.9 m/min,1 h/d,3d/w	4w/8w
80% VO_2_max		25.2 m/min,1 h/d,3d/w	4w/8w

### 2.3 Surgical protocol for ACLT of the rat knee

Due to their larger joint dimensions and thicker articular cartilage compared to mice, rats offer greater precision during surgical procedures for establishing PTOA models. Furthermore, rat models exhibit greater physiological and pathological similarity to humans than mouse models, enhancing their translational relevance and predictive validity. Therefore, we selected Sprague-Dawley (SD) rats to further investigate the potential regulatory role of ANTXR1 in cartilage homeostasis.

Twelve male SD rats (8 weeks old) were purchased from Beijing Huafukang Biotechnology Co. All rats were randomly divided into two groups: the Sham group (Sham group, n = 6) and the ACLT-induced OA model group (ACLT group, n = 6). The surgical procedure was briefly as follows: all surgical instruments were sterilized in an autoclave according to the set procedure. Rats were fasted 24 h before surgery and only water was provided. Rats were adequately anesthetized by inhalation of isoflurane and placed supine on the operating table. The skin was incised with a scalpel blade and the joint capsule of the knee was exposed. The ACL was further exposed by flexion of the rat knee. The ACLs of rats in the ACLT group were singed off using the tips of ophthalmic surgical scissors, whereas the ACLs of rats in the Sham group were left intact. Subsequently, the incisions were sutured layer by layer. Postoperative rats were protected from infection by intramuscular injection of penicillin.

### 2.4 Micro-CT scan

The knee joint was placed in paraformaldehyde fixative for 48 h. Subsequently, the knee joint was placed in the Skyscan 1276 scanning device for scanning. he scanning accuracy was 8 μm. At the end of the scan, the subchondral bone of the femur and tibia of the knee were reconstructed separately. The software CTan was used for the analysis of bone microstructural parameters, including bone mineral density (BMD), bone volume fraction (BV/TV), bone surface/bone volume (BS/BV), bone trabecular thickness (Tb.Th), and trabecular number (Tb.N), and bone trabecular segregation (Tb.Sp).

### 2.5 Hematoxylin-eosin (HE) staining and safranin O-Fast staining (saf-O/Fast Green)

After micro-CT scanning, 10% EDTA solution (Cat:E1171, Solarbio, China) was used to decalcify the knee bone tissue. Change the EDTA decalcification solution every 2–3 days. Complete decalcification was defined as the ability of a syringe tip to easily penetrate the cortical bone. It took approximately 3 weeks for mouse knee joints to complete decalcification, while it took approximately 4 weeks for rat knee joints. Subsequently, the knee joints were made into tissue wax blocks through the processes of dehydration, transparency, wax impregnation and embedding. Finally, the sections were cut using a wax slicer (section thickness of 4 μm). The paraffin sections were stained according to the standard instructions for HE staining and Saf-O/Fast Green staining. After histologic staining, the knee joint sections were photographed under a light microscope. The pathology of the knee joint tissue sections was scored separately by two independent researchers with experience in this field according to the criteria of Mankins score (as shown in Table 2) and OARSI score (as shown in Table 3). The mean value of the two observers was calculated as the final score.

**TABLE 2:** Mankin grading system.

Evaluation category	Score	Criteria
Cartilage Structure		
	0	Intact structure with smooth surface
	1	Slight surface irregularities or superficial fibrillation
	2	Fissures extending to the transitional zone
	3	Fissures extending into the radial zone
	4	Fissures extending to the calcified zone
	5	Complete cartilage erosion with subchondral bone exposure
Chondrocyte Changes		
	0	Normal cellularity and distribution
	1	Diffuse increase in cell density
	2	Presence of chondrocyte clusters (cell aggregation)
	3	Significant reduction in chondrocyte numbers
	4	Nearly complete absence of chondrocytes
Tidemark Integrity		
	0	Intact tidemark without disruptions
	1	Presence of multiple tidemarks
	2	Vascular invasion into the tidemark from the subchondral bone

**TABLE 3:** OARSI grading system.

Score	Criteria
0	Normal
0.5	Very Minimal Degeneration: Loss of t. Blue (or other cationic dye) stain (proteoglycan loss) without structural changes
1	Minimal Degeneration: Small surface to subsurface fibrillations without major loss of chondrocytes or cartilage matrix, may have small focal area of chondrocyte loss extending partial thickness over less than 5% of total surface
2	Mild Degeneration: Vertical clefts down to the layer immediately below the superficial layer with few extending deeper and some loss of surface matrix, or focal areas of chondrocyte/proteoglycan loss with good collagen preservation extending partial thickness over 5%–10% of the surface
3	Moderate Degeneration: Vertical clefts/erosion to the calcified cartilage extending over <25% of the articular surface, or focal areas of chondrocyte/proteoglycan loss with some collagen preservation extending full thickness over 10%–24% of the surface
4	Marked Degeneration: Vertical clefts/erosion to the calcified cartilage extending over 25%–50% of the articular surface, or focal areas of chondrocyte/proteoglycan loss with some collagen preservation extending full thickness over 25%–50% of the surface
5	Severe Degeneration: Vertical clefts/erosion to the calcified cartilage extending over 50%–75% of the articular surface, or focal areas of chondrocyte/proteoglycan loss with some collagen preservation extending full thickness over 50%–75% of the surface
6	Very Severe Degeneration: Vertical clefts/erosion to the calcified cartilage extending >75% of the articular surface, may be few areas of acellular collagen remaining

HE staining visualizes overall cartilage architecture, including chondrocyte distribution (lacunae), the tidemark, and the calcified/non-calcified cartilage boundary. Collagen fibers stain pink, while proteoglycans (PGs) appear as a homogeneous pale-pink matrix background, precluding their quantitative assessment. H&E enables evaluation of structural integrity, chondrocyte morphology, and inflammatory infiltration, facilitating identification of OA-associated lesions (e.g., surface defects, cellular abnormalities). In contrast, Saf-O/Fast Green selectively stains sulfated glycosaminoglycans (GAGs) within PGs bright red to orange-red. Staining intensity correlates directly with GAG density, with depleted regions showing reduced or absent signal. Fast Green counterstains collagen bluish-green, enhancing PGs/collagen contrast and revealing collagen network disruption. Although chondrocyte nuclei are weakly stained, cellular detail is less distinct than with HE. This method allows semi-quantitative assessment of PG loss, a critical early OA indicator. In our study, the severity of cartilage damage was evaluated using the widely adopted OARSI and Mankin scoring systems, both of which rely on Saf-O/Fast Green staining for OA severity grading. The OARSI scoring system exhibits superior sensitivity for depth-specific lesions and early-stage degeneration, whereas the Mankin system provides a comprehensive evaluation of cartilage structure, cellular activity, matrix metabolism, and calcified layer integrity, directly reflecting the proteoglycan synthesis/degradation equilibrium. This makes Mankin particularly suitable for assessing moderate-to-advanced OA. Furthermore, while OARSI serves as the gold standard for rodent ACLT models (PTOA), Mankin enables cross-species comparability. Employing both systems enhances evaluation reliability and strengthens conclusion robustness.

### 2.6 Bioinformatics analysis

The STRING website (https://string-db.org/) was utilized to retrieve and validate potential target proteins for ANTXR1. Briefly, upon accessing the homepage of the STRING website database, the search criteria were set as follows: (1) “protein name: ANTXR1”; (2) “Organism: mouse”; (3) “Minimum Required Interaction Score: Median confidence (0.400)”. After the system search was completed, the protein interactions graphs of ANTXR1 and LRP6 were displayed. Subsequently, molecular docking experiments were performed using the PDB files of Antxr1 (UniProt ID: Q9H6X2, Structure Identifier: 3N2N) and LRP6 (UniProt ID: O75581, Structure Identifier: 8FFE) downloaded from the UniProt database. The HDOCK website was used with default parameters to predict the binding sites between the two molecules. The top-scoring protein-protein docking conformation predicted by HDOCK was downloaded and visualized using the molecular visualization software PyMOL. Finally, the downloaded PDB files were uploaded to the PDBePISA website for conformational analysis, and the docking conformations were scored based on the output from the HDOCK website.

### 2.7 Immunofluorescence staining

Knee paraffin sections were sequentially deparaffinized, antigenically repaired overnight at 60°C using sodium citrate buffer (Beyotime, P0083), blocked with 3% BSA, incubated with primary antibody (anti-ANTXR1, abmart, TD13424S, Source-Rabbit, 1:400), incubated with secondary antibody (HRP-labeled goat anti-rabbit IgG-cy3), added with primary antibody (anti-LRP6, abmart, T58345, Source-Rabbit, 1: 100), incubated with secondary antibody (Alexa Fluor 488-labeled goat anti-rabbit IgG), restained the nuclei with DAPI, and sealed. Finally, photographs were taken under a Leica laser confocal microscope (Leica, TCS SP8, Germany). In this experiment, the primary antibody was incubated overnight at 4 °C. The secondary antibody was incubated at room temperature for approximately 1 h. ImageJ was used to perform statistical analysis of immunofluorescence images. In each sample, five ×400 magnification field images were acquired for fluorescence intensity statistics (relative expression analysis of fluorescence intensity of nuclei counted in the same field). The average fluorescence intensity of the five sets of data was used as representative data for the sample for intergroup difference analysis.

### 2.8 Data analysis

All data were analyzed using SPSS 20.0 software. Results are expressed as mean ± standard deviation ( $\bar{X}$ ± *S*). One way ANOVA and LSD method were used to compare the data between the groups. P < 0.05 indicated a significant difference.

## 3 Results

### 3.1 Treadmill exercise at different intensities promotes bone-cartilage health

**FIGURE 2:**
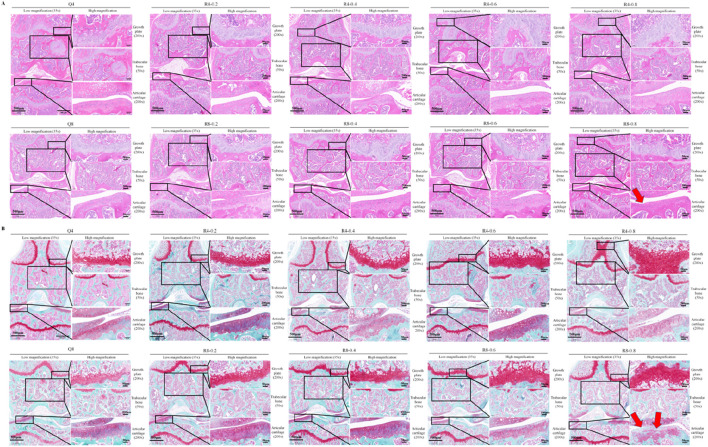
Histological staining of mouse knee joints. **(A)** HE staining of mouse knee joints; **(B)** Saffron O solid green staining of mouse knee joints; Red arrows indicate typical cartilage degeneration characteristics, Q4: 4w sedentary control group; R4-0.2: 4w treadmill exercise with 20% maximal oxygen uptake group; R4-0.4: 4w treadmill exercise with 40% maximal oxygen uptake group; R4-0.6: 4w treadmill exercise with 60% maximal oxygen uptake group; R4-0.8: 4w treadmill exercise with 80% maximal oxygen uptake group; Q8: 8w sedentary control group; R8-0.2: 8w treadmill exercise with 20% maximal oxygen uptake group; R8-0.4: 8w treadmill exercise with 40% maximal oxygen uptake group; R4-0.6: 4w treadmill exercise with 60% maximal oxygen uptake group; R8-0.8: 8w treadmill exercise with 80% maximal oxygen uptake group, n = 6).

Eight-week-old mice were used to perform 4w and 8w of mandatory moderate-intensity treadmill exercise to observe the effects of different intensities and durations of treadmill exercise on bone-cartilage health. As shown in Figures 2A,B, Histological staining results showed that cartilage growth plates were significantly thickened and the number of cancellous bone and articular chondrocytes were significantly increased in an intensity-dependent and time-dependent manner in treadmill training group compared to sedentary group, suggesting active osteogenic function. It is worth noting that high-intensity treadmill exercise (e.g., 80% of maximum oxygen consumption) may cause mild cartilage wear and partial cartilage cell death/cartilage loss (as indicated by the red arrows in Figure 2).

**FIGURE 3:**
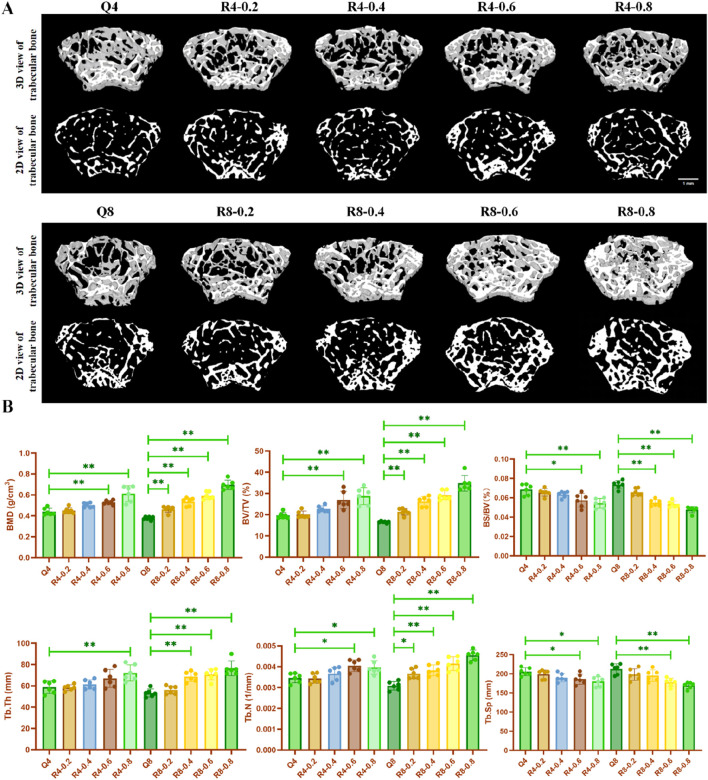
Reconstructed images and bone morphometric indices of mouse knee joints by micro-CT scanning. **(A)** Reconstructed images of mouse knee joints; **(B)** Altered bone morphometric indices of mouse knee joints, Q4: 4w sedentary control group; R4-0.2: 4w treadmill exercise with 20% maximal oxygen uptake group; R4-0.4: 4w treadmill exercise with 40% maximal oxygen uptake group; R4-0.6: 4w treadmill exercise with 60% maximal oxygen uptake group; R4-0.8: 4w treadmill exercise with 80% maximal oxygen uptake group; Q8: 8w sedentary control group; R8-0.2: 8w treadmill exercise with 20% maximal oxygen uptake group; R8-0.4: 8w treadmill exercise with 40% maximal oxygen uptake group; R4-0.6: 4w treadmill exercise with 60% maximal oxygen uptake group; R8-0.8: 8w treadmill exercise with 80% maximal oxygen uptake group, n = 6, *p < 0.05, **p < 0.05).

Articular cartilage and its underlying subchondral bone constitute a tightly coupled functional unit within the joint system. Cartilage anchors directly to the trabecular bone via the calcified layer, enabling shared mechanical load-bearing. Simultaneously, the vascular network within the trabecular spaces provides a critical channel for nutrient exchange to the calcified cartilage. The Osteoarthritis Research Society International (OARSI) guidelines emphasize that cartilage degeneration is invariably accompanied by subchondral bone remodeling, including sclerosis and cyst formation. Both subchondral bone alterations and cartilage degradation are defined as hallmarks of osteoarthritis (OA). Critically, evidence indicates that pathological changes in subchondral bone precede overt cartilage destruction (Bailey and Mansell, 1997; Pratt et al., 2012). The destruction of subchondral bone can lead to aberrant mechanical stress concentration on the overlying cartilage. This stress, in turn, may promote the release of catabolic mediators, further contributing to cartilage thinning and chondrocyte loss (Burr and Radin, 2003; He et al., 2020). Given this established mechanistic interdependence and the temporal precedence of bone changes, comprehensive assessment of subchondral bone is indispensable in studies of articular cartilage pathology. Therefore, our study employed micro-CT scanning to evaluate subchondral bone microstructure. Micro-CT scan results showed the same trend that treadmill exercise significantly increased BMD, BV/TV, BS/BV, Tb.Th, and Tb.N, and decreased Tb. Sp in the femur of mice. Furthermore, the bone formation-promoting effect of exercise was enhanced with increasing exercise intensity (as shown in Figures 3A,B). However, unlike the effect of promoting bone formation, the articular cartilage growth plate thinned and the number of chondrocytes was significantly reduced in the mice with 80% exercise intensity for 8 weeks relative to the other treadmill exercise groups, suggesting that the intensity of R8-0.8 maybe detrimental to cartilage homeostasis, which is consistent with our previous findings (Zhou et al., 2025).

### 3.2 Treadmill exercise inhibits ANTXR1 protein expression in cartilage

**FIGURE 4:**
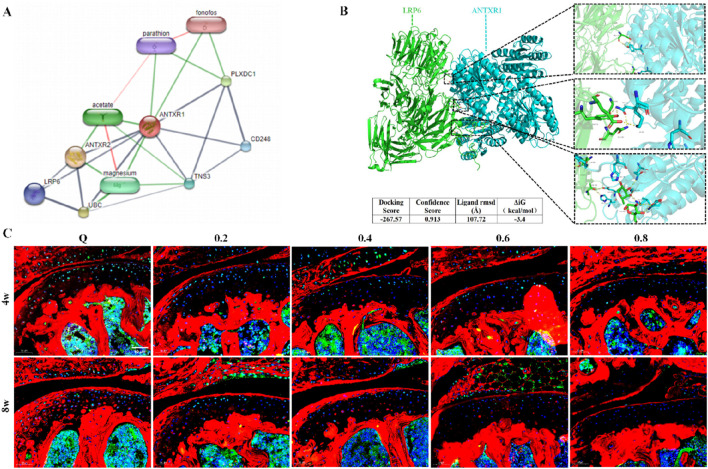
Treadmill exercise inhibits ANTXR1 protein expression in cartilage. **(A)** ANTXR1 target protein prediction by bioinformatics analysis; **(B)** Possible interaction of ANTXR1 and LRP6 proteins analyzed by molecular docking; **(C)** Expression of ANTXR1 and LRP6 proteins in mouse knee cartilage tissue by immunofluorescence staining, Q4: 4w sedentary control group; R4-0.2: 4w treadmill exercise with 20% maximal oxygen uptake group; R4-0.4: 4w treadmill exercise with 40% maximal oxygen uptake group; R4-0.6: 4w treadmill exercise with 60% maximal oxygen uptake group; R4-0.8: 4w treadmill exercise with 80% maximal oxygen uptake group; Q8: 8w sedentary control group; R8-0.2: 8w treadmill exercise with 20% maximal oxygen uptake group; R8-0.4: 8w treadmill exercise with 40% maximal oxygen uptake group; R4-0.6: 4w treadmill exercise with 60% maximal oxygen uptake group; R8-0.8: 8w treadmill exercise with 80% maximal oxygen uptake group, n = 6).

Previous studies in our group showed that ANTXR1 can participate in the regulation of bone formation (Liu et al., 2024). However whether ANTXR1 is involved in cartilage homeostasis is not completely clear. First, bioinformatics analysis and molecular docking techniques were used to identify the target protein of ANTXR1 as LRP6 (Figures 4A,B). Subsequently, the expression of the above two proteins in the knee joints of mice in each group was examined by immunofluorescence staining. As shown in Figure 4C, compared with the sedentary group, both ANTXR1 and LRP6 protein expression was lower in the knee cartilage of mice in the 4w and 8w exercise intervention groups, and the protein expression gradually decreased with the increase of exercise intensity. It is noteworthy that ANTXR1 and LRP6 showed significantly high expression in bone tissues compared to cartilage tissues. This may be the reason why few previous studies have focused on the potential role of ANTXR1 in cartilage, possibly due to the low expression of ANTXR1 in healthy cartilage and thus neglected.

### 3.3 ANTXR1 may be involved in regulating PTOA progression

**FIGURE 5:**
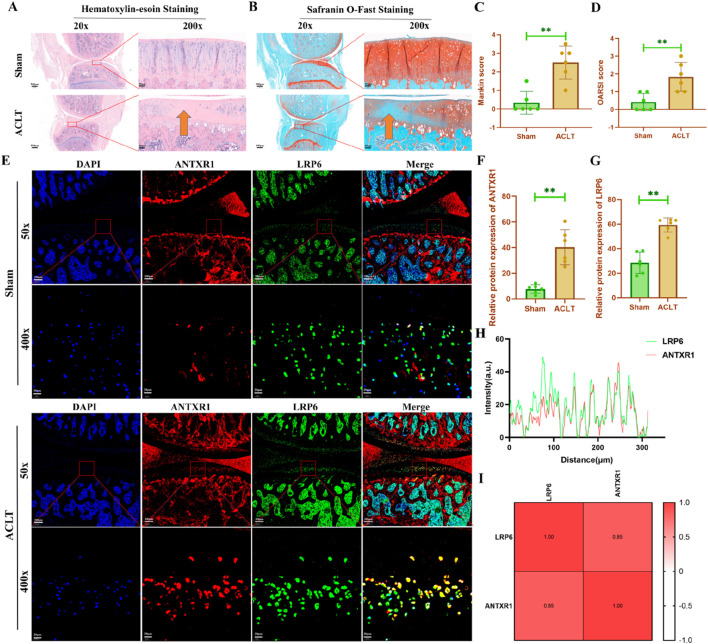
Histological staining of rat knee joints. **(A)** HE staining of rat knee joints; **(B)** saffron O solid green staining of rat knee joints; **(C)** Mankin score of rat knee cartilage; **(D)** OARSI score of rat knee cartilage; **(E-G)** Expression of ANTXR1 and LRP6 proteins in rat knee cartilage tissue by immunofluorescence staining; **(H, I)** Correlation analysis of ANTXR1 and LRP6 proteins, based on immunofluorescence staining. Orange arrows indicate typical cartilage degeneration characteristics, Q4: 4w sedentary control group; R4-0.2: 4w treadmill exercise with 20% maximal oxygen uptake group; R4-0.4: 4w treadmill exercise with 40% maximal oxygen uptake group; R4-0.6: 4w treadmill exercise with 60% maximal oxygen uptake group; R4-0.8: 4w treadmill exercise with 80% maximal oxygen uptake group; Q8: 8w sedentary control group; R8-0.2: 8w treadmill exercise with 20% maximal oxygen uptake group; R8-0.4: 8w treadmill exercise with 40% maximal oxygen uptake group; R4-0.6: 4w treadmill exercise with 60% maximal oxygen uptake group; R8-0.8: 8w treadmill exercise with 80% maximal oxygen uptake group, n = 6, **p < 0.05).

To further explore the potential regulatory role of ANTXR1 in cartilage homeostasis, the ACLT surgery was used to construct an animal model of OA to investigate whether ANTXR1 is involved in regulating the initiation and progression of OA. Our previous study demonstrated that moderate-intensity exercise is effective in alleviating OA (Zhou et al., 2021). Based on the regulatory effect of exercise on cartilage ANTXR1 expression in this study, we speculate that ANTXR1 may be involved in regulating PTOA. As shown in Figure 5A, HE staining of rat knee joints showed that the articular surface of the knee joints of rats in the ACLT group was rough, with a few irregular fissures and a reduced number of chondrocytes on the surface, compared with that of the Sham group. Mankin score showed statistically significant differences in the above cartilage pathologic changes (Figure 5C). Similarly, as shown in Figure 5B, the results of saffron O solid green staining showed smooth knee joint surfaces in the Sham group of mice, whereas significant cartilage loss was present in the ACLT group of mice. The comparison of OARSI score results between the two groups was significantly different (Figure 5D). In addition, to further validate the role of ANTXR1 in cartilage homeostasis, we examined the expression of ANTXR1 in PTOA cartilage tissues for the first time. The results revealed that ANTXR1 showed high expression in cartilage tissues of mice in the ACLT group. In addition, the target protein of ANTXR1, LRP6, showed the same trend (Figures 5E–G). The above results suggest that ANTXR1 may be involved in the initiation and progression of PTOA.

## 4 Discussion

This study employed two intervention durations (4 weeks and 8 weeks) across four treadmill exercise intensities (20%, 40%, 60%, and 80% of VO_2_max). The protocol was adapted from established studies evaluating the anti-inflammatory, matrix-synthesizing, and chondroprotective effects of treadmill exercise in instability-induced OA models (typically 12–18 m/min, 30–60 min/day, 3–7 days/week for 4–8 weeks) (Coughlin and Kennedy, 2016; Nam et al., 2011). These intensity ranges align with our measured VO_2_max values. Specifically, 80% VO_2_max corresponded to a running speed of 25.2 m/min, consistent with high-intensity protocols used in prior research (Li et al., 2017). Furthermore, evidence indicates that a 4-week intervention captures early-stage adaptive responses in cartilage, while sustained mechanical loading over 8 weeks drives structural alterations, such as proteoglycan reduction and subchondral bone remodeling (Hao et al., 2021). The results showed that moderate-intensity exercise (40%–60% VO_2_max) contributed to the promotion and maintenance of cartilage homeostasis, whereas high-intensity prolonged exercise (80% VO_2_max for 8w) may cause mild damage to cartilage, which may be related to the induction of oxidative stress or inflammatory factors by mechanical overload. This result is consistent with previous reports that low-intensity exercise promotes chondrocyte autophagy and ECM synthesis to improve cartilage homeostasis, whereas high-intensity exercise activates chondrocyte pyroptosis by inducing ROS accumulation (Zhou et al., 2020). Moderate mechanical stress may maintain the balance between ECM synthesis and degradation by activating the PI3K/Akt/mTOR pathway to promote chondrocyte autophagy and removal of damaged organelles (Caramés et al., 2012).

In contrast to the detrimental effects on cartilage, micro-CT analysis revealed a progressive increase in exercise-induced bone formation with higher intensities. While high-intensity treadmill exercise negatively impacted cartilage, it had no deleterious effects on bone tissue. This divergence likely reflects distinct mechanotransduction mechanisms: bone tissue adapts to mechanical stress via osteoblast-mediated mineralization, whereas chondrocytes exhibit heightened vulnerability to sustained high-magnitude loading.

Our previous study demonstrated that the mechanosensitive protein ANTXR1 impairs bone formation by inhibiting Wnt/β-catenin signaling (Liu et al., 2024). In this study, we observed a progressive decrease in ANTXR1 expression in mouse articular cartilage with increasing exercise intensity, suggesting its detrimental role in joint cartilage homeostasis. Although exercise intervention regulates ANTXR1 protein expression in an intensity- and time-dependent manner, the underlying mechanisms remain unclear. To investigate ANTXR1’s function in cartilage maintenance, we established a PTOA model via ACLT surgery. Notably, a dual-species approach (murine/rat) was employed: Mice enabled high-compliance multi-timepoint/dose treadmill interventions, while rats provided enhanced modeling precision and clinical translatability for mechanistic studies. Bioinformatics analysis identified LRP6 as a potential target of ANTXR1, which was corroborated by immunofluorescence assays showing co-upregulation of both proteins in degenerated cartilage of ACLT-induced PTOA mice. As a core mechanotransduction molecule, ANTXR1 directly responds to mechanical stress and pathological processes (Feng et al., 2023), aligning with our finding that exercise suppresses LRP6 expression in cartilage. In addition, studies have shown that LRP6 is also a mechanosensitive protein critical for transducing mechanical stimuli (e.g., tensile stress, fluid shear stress). Previous studies reported that LRP6 can participate in the modulation of the Wnt/β-catenin pathway (Ng et al., 2023), which is one of the key signaling pathways in the regulation of cartilage homeostasis in OA (Yao et al., 2023). These results imply that exercise may mitigate osteoarthritis by inhibiting the ANTXR1/LRP6 axis—a hypothesis requiring further validation. Future studies also need to pay more attention to the ANTXR1/LRP6 downstream signaling cascade, which can help to deeply understand the potential role of ANTXR1/LRP6 signaling in OA progression.

Notably, the high expression of ANTXR1 in bone tissue contrasts with the low expression in cartilage. Future studies could consider clarifying whether bone-derived ANTXR1 affects cartilage metabolism through the paracrine pathway through conditional knockout animal models (Chen et al., 2025), thus providing a more precise strategy for OA-targeted intervention.

The limitation of this study is mainly due to the insufficient direct validation of the ANTXR1 regulatory mechanism. Future studies could incorporate chondrocyte knockdown/overexpression of ANTXR1 and construct cartilage-specific ANTXR1 knockout mice to delve into the specific molecular mechanisms by which ANTXR1 alleviates PTOA. In addition, the evidence of bioinformatics predicting the interaction of ANTXR1 with LRP6 was insufficient. The combination of techniques such as immunoprecipitation is needed to provide more sufficient evidence in the future.

## 5 Conclusion and perspectives

In the present study, we found that mechanical stress significantly inhibited the expression of ANTXR1 and LRP6 proteins in mouse cartilage in an intensity-dependent and time-dependent manner. In addition, we found that ANTXR1 protein was significantly upregulated in cartilage tissues in the ACLT-induced PTOA rat model, suggesting that ANTXR1 protein may be involved in regulating the initiation and progression of PTOA. Overall, this study elucidated that the mechanosensitive protein ANTXR1 may be involved in the regulation of maintaining cartilage homeostasis, which provides a theoretical basis for optimizing exercise intervention strategies. Combined with the intensity-dependent and time-dependent nature of exercise interventions, it may be possible to achieve precision treatment of PTOA through a combined exercise and drug strategy.

## Data Availability

The original contributions presented in the study are included in the article/Supplementary Material, further inquiries can be directed to the corresponding authors.
