# Supplementary material for: The mechanosensitive protein ANTXR1 is involved in maintaining cartilage homeostasis in post-traumatic osteoarthritis
Source: Front Cell Dev Biol. 2025 Sep 3;13:1625333. doi: 10.3389/fcell.2025.1625333 (PMC12440867; doi:10.3389/fcell.2025.1625333)
Supplement: Supplementary file 1 [file DataSheet1.zip › Supplementary Files/Supplementary Figures and Tables.docx]

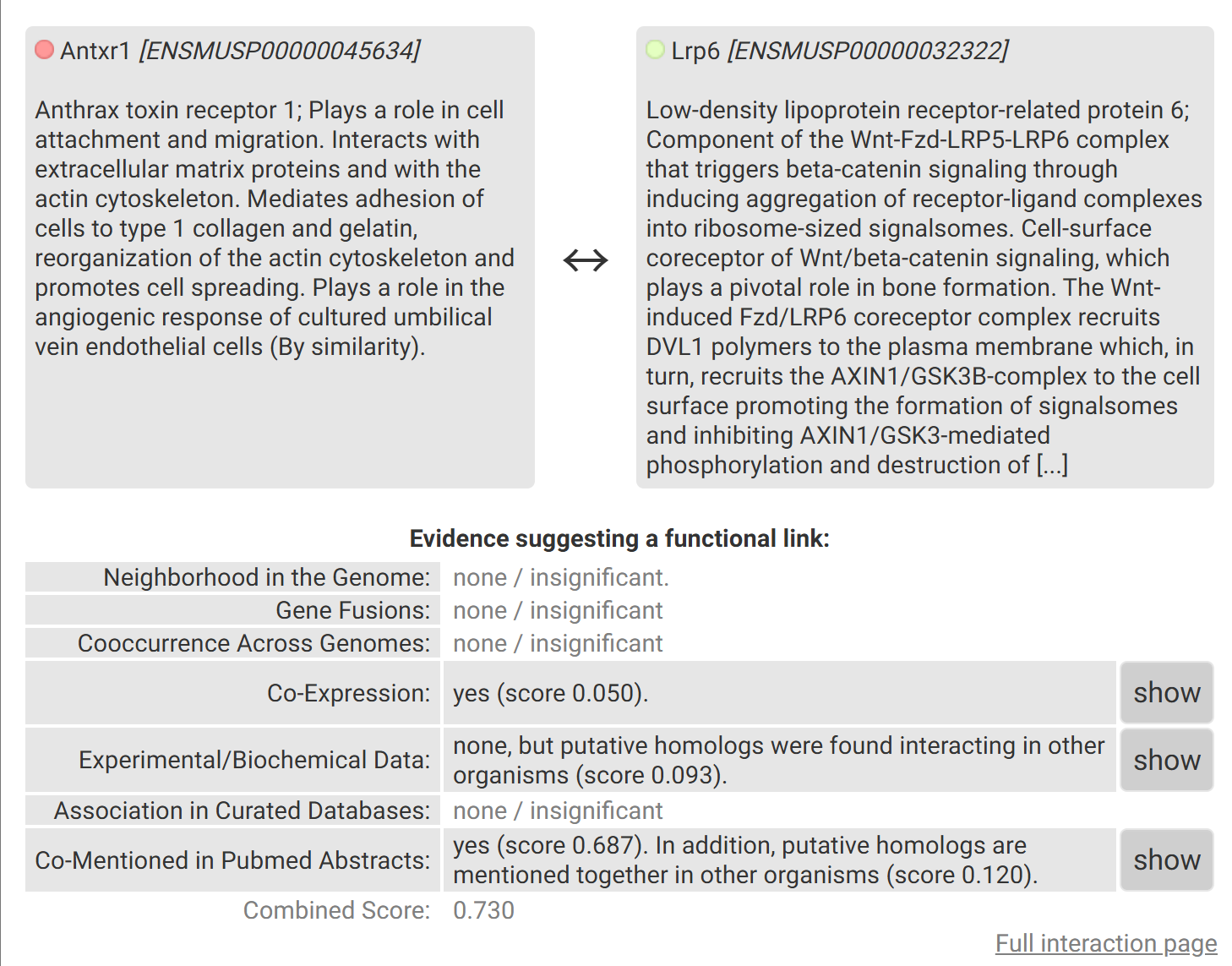


**Figure S1 The original data graph of the target protein prediction results of ANTXR1**


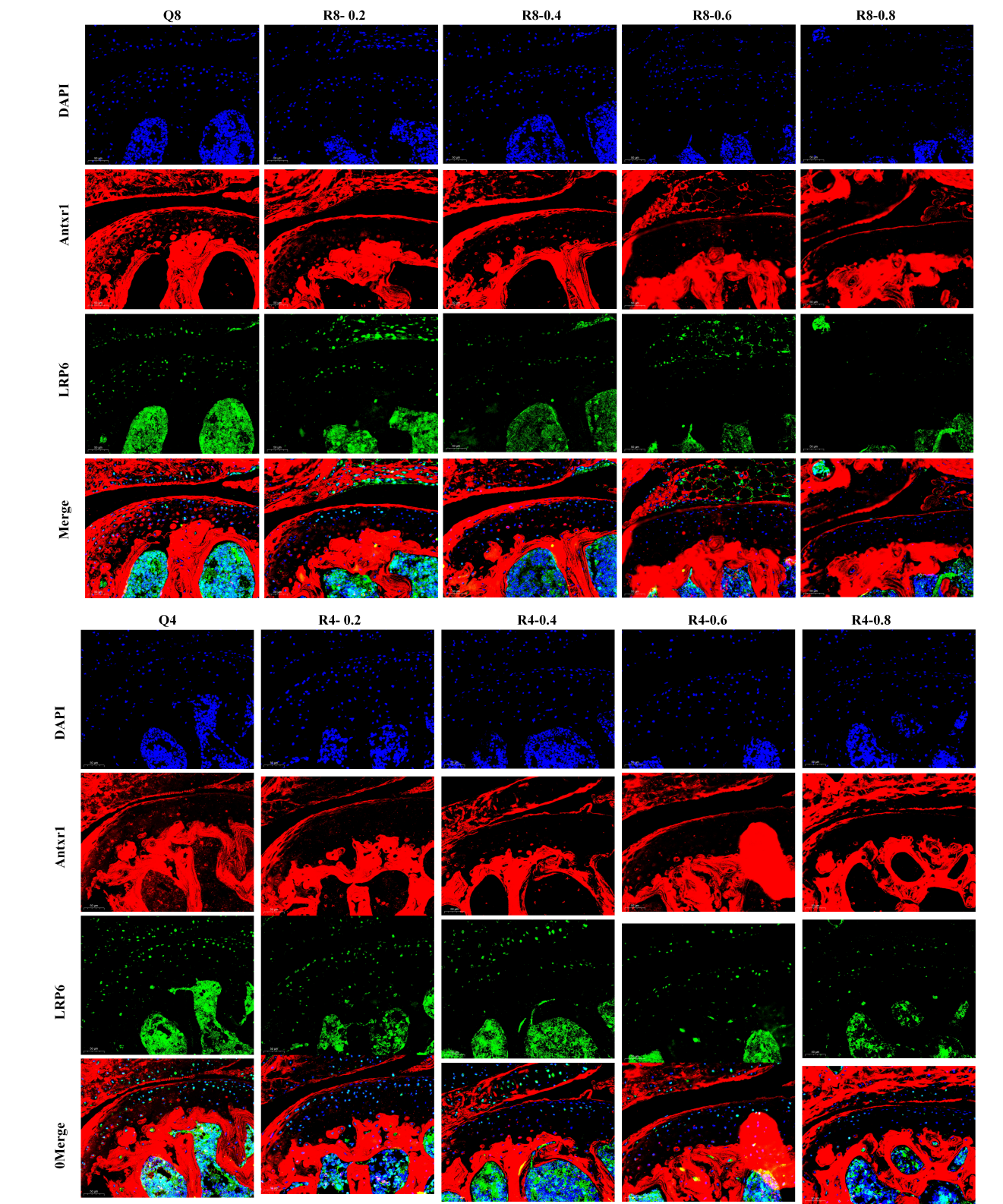


**Figure S2 Dual-color fluorescence images of the ANTXR1 and LRP6 proteins (in a single fluorescence channel)**

**Table S1. OARSI Grading System**

| 0 | Normal |
| --- | --- |
| 0.5 | Very Minimal Degeneration: Loss of t. blue (or other cationic dye) stain (proteoglycan loss) without structural changes |
| 1 | Minimal Degeneration: Small surface to subsurface fibrillations without major loss of chondrocytes or cartilage matrix, may have small focal area of chondrocyte loss extending partial thickness over less than 5% of total surface |
| 2 | Mild Degeneration: Vertical clefts down to the layer immediately below the superficial layer with few extending deeper and some loss of surface matrix, or focal areas of chondrocyte/proteoglycan loss with good collagen preservation extending partial thickness over 5 to 10% of the surface |
| 3 | Moderate Degeneration: Vertical clefts/erosion to the calcified cartilage extending over <25% of the articular surface, or focal areas of chondrocyte/proteoglycan loss with some collagen preservation extending full thickness over 10 to 24% of the surface |
| 4 | Marked Degeneration: Vertical clefts/erosion to the calcified cartilage extending over 25 to 50% of the articular surface, or focal areas of chondrocyte/proteoglycan loss with some collagen preservation extending full thickness over 25 to 50% of the surface |
| 5 | Severe Degeneration: Vertical clefts/erosion to the calcified cartilage extending over 50 to 75% of the articular surface, or focal areas of chondrocyte/proteoglycan loss with some collagen preservation extending full thickness over 50 to 75% of the surface |
| 6 | Very Severe Degeneration: Vertical clefts/erosion to the calcified cartilage extending >75% of the articular surface, may be few areas of acellular collagen remaining |

**Table S2. Mankin Grading System**

| Evaluation Category | Score | Criteria |
| --- | --- | --- |
| Cartilage Structure |  |  |
|  | 0 | Intact structure with smooth surface. |
|  | 1 | Slight surface irregularities or superficial fibrillation. |
|  | 2 | Fissures extending to the transitional zone. |
|  | 3 | Fissures extending into the radial zone. |
|  | 4 | Fissures extending to the calcified zone. |
|  | 5 | Complete cartilage erosion with subchondral bone exposure. |
| Chondrocyte Changes |  |  |
|  | 0 | Normal cellularity and distribution. |
|  | 1 | Diffuse increase in cell density. |
|  | 2 | Presence of chondrocyte clusters (cell aggregation). |
|  | 3 | Significant reduction in chondrocyte numbers. |
|  | 4 | Nearly complete absence of chondrocytes. |
| Tidemark Integrity |  |  |
|  | 0 | Intact tidemark without disruptions. |
|  | 1 | Presence of multiple tidemarks. |
|  | 2 | Vascular invasion into the tidemark from the subchondral bone. |

**Table S3 Potential correlations between the immunofluorescence data (ANTXRI and LRP6) and the histological scoring results (OASRI and MANKIN).**

|  | ANTXRI | LRP6 |
| --- | --- | --- |
| OARSI | -0.841^*^ | 0 |
| MANKIN | 0.203 | 0.632 |
| ** p<0.05* |  |  |
